# Juvenile Psammomatoid Ossifying Fibroma (JPOF) of Proximal Radius: A Rare Entity

**DOI:** 10.2174/1874325001711010583

**Published:** 2017-07-28

**Authors:** Jagadish Prabhu, Veena Nagaraj, Iftikhar Ahmed Mukhtar

**Affiliations:** 1 Department of Orthopedics, Bahrain Defence Force Hospital - Royal Medical Services, Riffa, Kingdom of Bahrain.; 2 Department of Pathology, Bahrain Defence Force Hospital - Royal Medical Services, Riffa, Kingdom of Bahrain.; 3 Bahrain Defence Force Hospital - Royal Medical Services, Riffa, Kingdom of Bahrain.

**Keywords:** Ossifying fibroma, Psammomatoid, Juvenile ossifying fibroma

## Abstract

**Background:**

Juvenile psammomatoid ossifying fibroma (JPOF) is a rare fibro-osseous lesion that usually occurs in the facial bones. The pathognomonic histopathologic feature is the presence of spherical ossicles, which are similar to psammoma bodies. It is considered to be a unique lesion because of its reported tendency to occur in children and adolescents and its tendency for locally aggressive growth. Because this lesion is aggressive in nature with high recurrence rate, early detection and complete surgical excision are essential.

**Case Report:**

Herein, we present a case of 11 year old girl, who presented to our clinic with history of gradual onset pain around right elbow associated with limitation of right forearm rotation for 3 months. After getting informed consent, we performed needle biopsy and histopathologically it was diagnosed as Psammomatoid type juvenile ossifying fibroma (JPOF) of proximal radius. We performed extensive debridement, curettage of entire cystic lesion involving the proximal radius and filled the cavity with allograft bone granules and intramedullary titanium elastic nail through fibular strut graft, bridging the cystic lesion.

**Conclusion:**

As far as our knowledge there is no reported case of psammomatoid type of juvenile ossifying fibroma of proximal radius. We believe that such tumors in long bones are aggressive and have tendency for recurrence. Definitive diagnosis is utmost important for proper planning, surgical excision and reconstruction of long bones and need regular follow up to look for any recurrence or malignant transformation.

## 1 INTRODUCTION

Juvenile ossifying fibroma (JOF) is a fibro-osseous neoplasm described as an actively growing lesion consisting of a cell-rich fibrous stroma, containing bands of cellular osteoid without osteoblastic lining, together with trabeculae of more typical woven bone. El-Mofty (2002) described two variants of Juvenile ossifying fibroma (JOF), namely Juvenile psammomatoid ossifying fibroma (JPOF) and Juvenile trabecular ossifying fibroma (JTOF) [1-6] Radiographically both exhibit a mixture of radiolucent and radiodense areas with thin sclerotic rims that may be incomplete [4]. Histopathologically JPOF forms concentric laminated, spherical ossicles, basophilic center with peripheral eosinophilic osteoid rims [6]. Complete clinical examination, imaging and histopathological features are required to label the diagnosis of JPOF. Because of unpredictable rapid and progressive growth, the management and the prognosis are uncertain.

## CASE REPORT

**Fig. (1):**
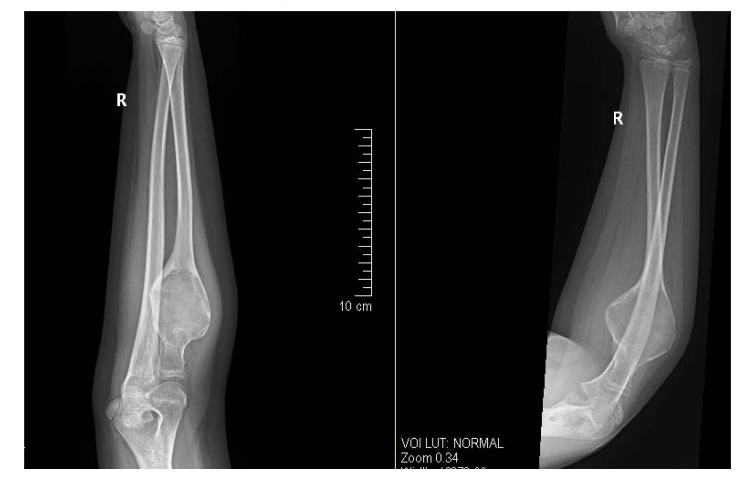
Pre-operative radiograph showing expansile osteolytic lesion of proximal radius, benign in nature with differential diagnosis of aneurysmal bone cyst or simple bone cyst.

**Fig. (2):**
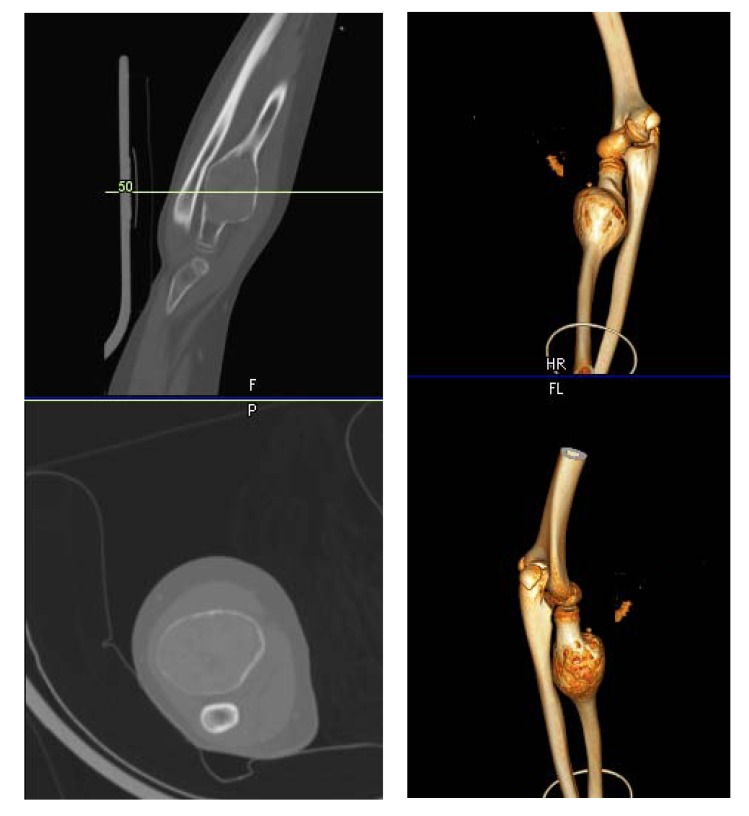
Pre-operative CT scan images showing markedly expansile lytic lesion measuring 4.5 cm X 3.8 cm in transverse diameter at proximal radial shaft with thinning of cortex, short trasitional zone, without periosteal reaction or surrounding soft tissue involvement.

Herein, we present a case of 11 year old girl, who presented to our clinic with history of gradual onset pain around right elbow associated with limitation of right forearm rotation for last 3 months. Initially she was treated at her native place with some analgesic medications. Since pain and limitation of her activities in school was increasing, she approached our hospital for further investigation and treatment. She didn’t give any history of weight loss or any other constitutional symptoms. On detailed examination, we found that there was diffused swelling around dorsofeatures were suggestive oradial aspect of proximal right forearm with local tenderness over proximal radius. Active and passive flexion was 0-100* and rotation was from 30* supination to 20* pronation. Plain radiograph showed huge cystic bony lesion with multiple septae (Fig. **1**) and CT scan images showed markedly expansile lytic lesion measuring 4.5 cm X 3.8 cm in transverse diameter at proximal radial shaft with thinning of cortex, short trasitional zone, without periosteal reaction or surrounding soft tissue involvement (Figs. **2A**, **B**).

**Fig. (3):**
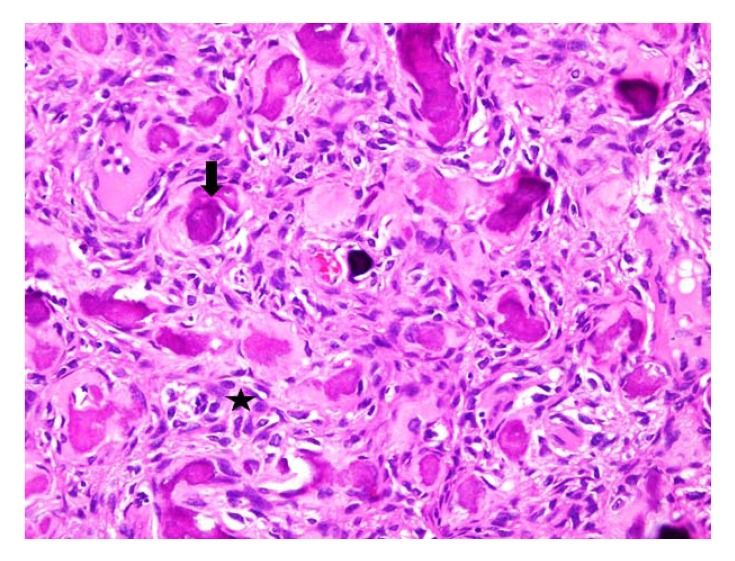
Numerous spindle-shaped cells arranged in fascicular storiform pattern (asterix) with irregular strands of trabeculae with plump osteoblast, spheroidal ossicles with basophilic center and eosinophilic periphery resembling psammoma-like bodies (arrow). H & E X 40X.

**Fig. (4):**
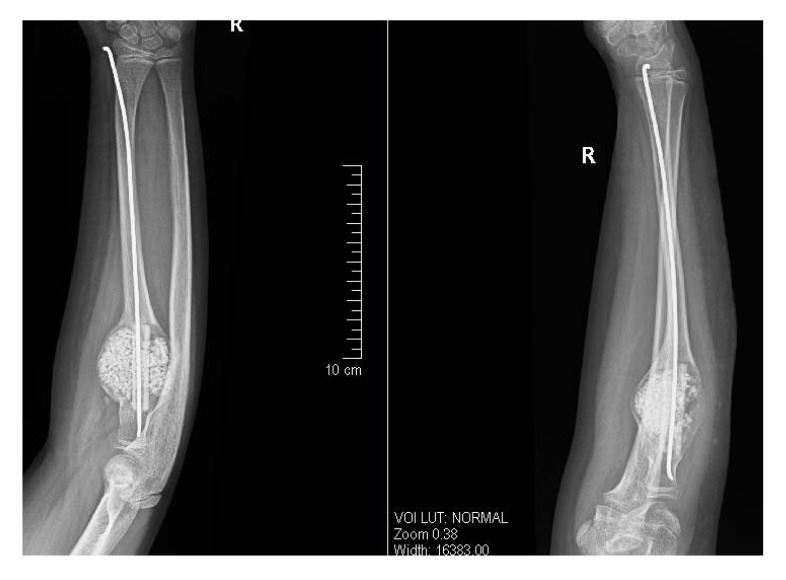
Immediate post op radiograph showing completely curetted cystic bony lesion with filled allograft granules, bridging fibular strut graft and transfixing intra-medullary titanium elastic nail insitu.

**Fig. (5):**
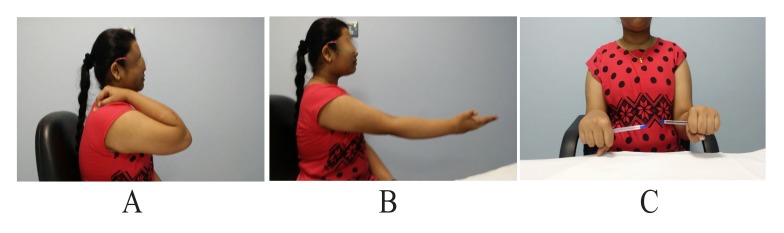
One year follow up clinical pictures showing range of motion of elbow joint movement.

**Fig. (6):**
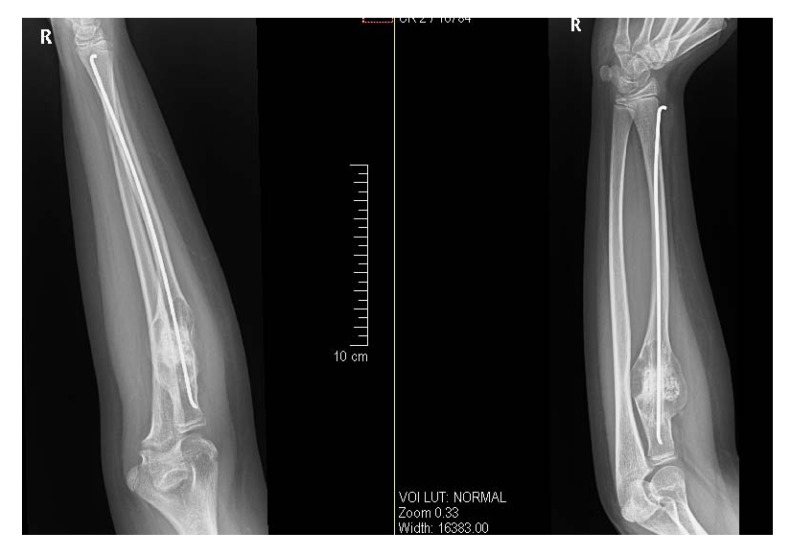
One year follow-up post-operative radiograph showing incorporation of allograft and fibular strut graft with bony union and without any signs of recurrence of tumor.

After getting informed consent, we performed needle biopsy and histopathologically it was reported as Psammomatoid type juvenile ossifying fibroma (JPOF) of proximal radius (Fig. **3**). The overall clinical, imaging and histopathological features were suggestive of non-aggressive type of JPOF. Considering this diagnosis, we considered extensive curettage and fibular strut graft interposition rather than enbloc excision of the tumor. Through Kaplan’s dorsal approach, we performed extensive debridement and curettage of entire cystic lesion involving the proximal radius. We broke the tumor cortex, removing the mechanical block involving the proximal radioulanr joint. We later filled the cavity with allograft bone granules and intramedullary titanium elastic nail through autologous fibular strut graft, bridging the cystic lesion (Fig. **4**). On table there was significant increase in range of rotational movement of forearm. She was put on above elbow back slab for 4 weeks and followed by gradual range of motion and strengthening exercises. She was on regular follow up thereafter. At 18 months follow up, she was symptom free with almost full range of active elbow motion, without any functional limitation (Figs. **5A**, **B**, **C**) and follow up radiograph shows incorporation of allograft and fibular strut graft without any signs of recurrence (Fig. **6**).

## DISCUSSION

The radiographic features of JPOF can resemble that of other lesions, such as fibrous dysplasia and cemento-ossifying fibroma, aneurysmal bone cyst (ABC), Osteogenic sarcoma and Osteoblastoma [7-9]. JPOF is not capsulated but is separated from surrounding bone by a radiopaque border [8, 10-11] and this finding can help in differentiating it from fibrous dysplasia [8]. JPOF usually presents as round well defined, sometimes corticated osteolytic lesion with a cystic appearance, with sclerotic changes being evident as ground glass appearance, while JTOF appears as a ground glass multilocular honeycomb [11]. Aggressiveness of the lesion with marked destruction of adjacent structures and presence of osteogenic elements make it sometimes difficult to differentiate from osteogenic sarcoma radiographically; the lack of periosteal reaction in JOF may help in differentiation [12].

Histopathological differential diagnoses of JPOF include cementum-forming neoplasms, which include cementifying fibromas, cementoblastoma, and familial gigantiform cementoma [13]. Cementifying fibromas most frequently occur in the third and fourth decades, primarily affecting female patients. Among the histologic findings observed in cementum forming lesions are structures called cementum ‘droplets’ or ‘cementicles’ [14] which are characterized by the presence of numerous round to oval mineralized spherules. JPOF and JTOF have been distinguished on the basis of their histopathological features, site, and age of occurrence. JPOF commonly affects patients older than JTOF. Histologically, the juvenile variants share a similar stroma but JPOF is characterized by innumerable small spherical or lamellated ossicles resembling psammoma bodies, while JTOF contains trabeculae of fibrillary osteoid and woven bone [15, 16].

The larger and more aggressive lesions require resection with 5 mm margins. Close clinical and radiological follow up is required for JPOF. Recurrence after surgical management of JPOF is common and is reported to range from 30% to 56% [17]. Longstanding lesions may show significant cortical destruction and periosteal elevation [18], which leads to increased risk of recurrence. Although, there is no reported case of malignant transformation, rapidly growing lesions should raise suspicion. Definitive diagnosis is utmost important for proper planning, surgical excision and reconstruction of long bones and need regular follow up to look for any recurrence or malignant transformation.

## CONCLUSION

According to our knowledge, there is no reported case of psammomatoid type of juvenile ossifying fibroma of proximal radius. There are several case reports of such tumor involving maxilla-fascial bones. But in our opinion, the behavior of such tumors is similar and so the diagnosis and treatment principles remain to be same. We believe that management of JPOF should be based on location, type, variant, aggressiveness, duration of presentation, and involvement of surrounding soft tissue. In our opinion, non-aggressive JPOF based on clinical, imaging, histopathological features can be treated by extensive intra-lesional curettage and local excision, but aggressive types should be treated by en-bloc or radical excision to prevent recurrences. At the same time, we also understand that we need to follow up this case on long term basis to make final conclusion regarding our mode of treatment for such tumor. Our main aim was to report this rare tumor involving the upper limb bone rather than to give surgical management protocol.
